# Topological spin and valley pumping in silicene

**DOI:** 10.1038/srep31325

**Published:** 2016-08-10

**Authors:** Wei Luo, L. Sheng, B. G. Wang, D. Y. Xing

**Affiliations:** 1 National Laboratory of Solid State Microstructures and Department of Physics, Nanjing University, Nanjing, 210093, China; 2 Collaborative Innovation Center of Advanced Microstructures, Nanjing University, Nanjing, 210093, China

## Abstract

We propose to realize adiabatic topological spin and valley pumping by using silicene, subject to the modulation of an in-plane *ac* electric field with amplitude *Ey* and a vertical electric field consisting of an electrostatic component and an *ac* component with amplitudes 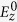 and 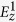. By tuning 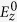 and 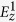, topological valley pumping or spin-valley pumping can be achieved. The low-noise valley and spin currents generated can be useful in valleytronic and spintronic applications. Our work also demonstrates that bulk topological spin or valley pumping is a general characteristic effect of two-dimensional topological insulators, irrelevant to the edge state physics.

Topological transport phenomena are generally protected by certain topological invariants, and exhibit universal properties that are immune to impurity scattering and insensitive to material details. Since the discovery of the integer quantum Hall (IQH) effect in two-dimensional (2D) electron systems^1^ in 1980, the first example of the topological transport phenomena, the fascinating characteristics of topological transport continue to be the primary focus of more and more research activities. Laughlin interpreted the precise integer quantization of the Hall conductivity in units of *e*^2^/*h* in the IQH effect in terms of an adiabatic quantum charge pump^2^. Thouless, Kohmoto, Nightingale, and Nijs established a relation between the quantized Hall conductivity and a topological invariant^3^, namely, the TKNN number or the Chern number. Thouless and Niu further related the amount of charge pumped in a charge pump to the Chern number^4^.

In recent years, the quantum spin Hall (QSH) effect, a spin analogue of the IQH effect, was proposed theoretically^5^,^6^, and realized experimentally in HgTe quantum wells^7^ and InAs/GaSb bilayers^8^. A QSH system, also called a 2D topological insulator (TI)^9^,^10^, is insulating in the bulk with a pair of gapless helical edge states^11^ at the sample boundary. In the ideal case, where the electron spin is conserved, a QSH system can be viewed as two independent IQH systems without Landau levels^12^, so that the topological properties of the system can be described by the opposite Chern numbers of the two spin species. In general, when the electron spin is not conserved, unconventional topological invariants, either the *Z*_2_ index^13^ or spin Chern numbers^14^,^15^,^16^, are needed to describe the QSH systems. The time-reversal symmetry is considered to be a prerequisite for the QSH effect, which protects both the *Z*_2_ index and gapless nature of the edge states. However, based upon the spin Chern numbers, it was shown that the bulk topological properties remain intact even when the time-reversal symmetry is broken. This finding evokes the interest to pursue direct investigation and utilization of the robust topological properties of the TIs, besides using their symmetry-protected gapless edge states, which are more fragile in realistic environments.

Recently, Chen *et al*. proposed that a spin Chern pumping effect from the bulk of the 2D TI, a HgTe quantum well, can be realized by using time-dependent dual gate voltages and an in-plane *ac* electric field^17^, which paves a way for direct investigation and utilization of the bulk topological properties of the TIs. The work of Chen *et al*. is a generalization of the earlier proposals of topological spin pumps^18^,^19^,^20^,^21^,^22^, based upon 1D abstract models, to a realistic 2D TI material. The spin Chern pump is a full spin analogue to the Thouless charge pump, in the sense that it is driven by topological invariants alone, without relying on any symmetries. For example, it has been shown that magnetic impurities breaking both spin conservation and time-reversal symmetry only modify the amount of spin pumped per cycle in a perturbative manner^17^,^22^, being essentially distinct from the QSH effect. Wan and Fischer suggested to realize a topological valley resonance effect in graphene by using the time-dependent lattice vibration of optical phonon modes, which can pump out a noiseless and quantized valley current flowing into graphene leads^23^. This topological valley resonance effect is intimately related to the spin or valley Chern pumping, as it is solely attributable to the valley Chern numbers, independent of the time-reversal symmetry^23^.

Silicene, the cousin of graphene, is a monolayer of silicon atoms instead of carbon atoms on a 2D honeycomb lattice. Recently, this material has been experimentally synthesized^24^,^25^,^26^ and theoretically explored^27^,^28^,^29^,^30^. Similar to graphene, the energy spectrum of silicene has two Dirac valleys, around the *K* and *K*′ points sited at opposite corners of the hexagonal Brillouin zone. Silicene has a much larger spin-orbit gap than graphene, favoring the QSH effect. As another prominent property distinguishing it from graphene, silicene has a buckled lattice structure, which allows us to control the Dirac masses at *K* and *K*′ points independently, by applying an external vertical electric field^29^,^31^. This property also makes silicene be a natural candidate for valleytronics^32^,^33^,^34^.

In this paper, we propose an experimental scheme to achieve topological spin and valley pumping by applying in silicene an in-plane *ac* electric field with amplitude *E*_*y*_ and a vertical electric field comprising an electrostatic component and an *ac* component with amplitudes 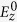 and 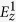. The present proposal is more practicable experimentally than the previous one^17^, because applying a vertical electric field in silicene has been much better understood^29^,^31^ and is more practical than applying dual gate voltages in HgTe quantum wells. By using the spin-valley Chern numbers, it is shown that the system can be in the pure valley pumping regime, mixed spin and valley pumping regime, or trivial pumping regime, depending on the strengths 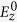 and 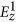 of the perpendicular electric field. The total amount of valley or spin quanta pumped per cycle, calculated from the scattering matrix formula, is fully consistent with the spin-valley Chern number description. It is proportional to the cross-section of the sample, and insensitive to the material parameters, a clear evidence that the pumping is a bulk topological effect, irrelevant to the edge states.

## Results

### Model Hamiltoinan

**Figure 1:**
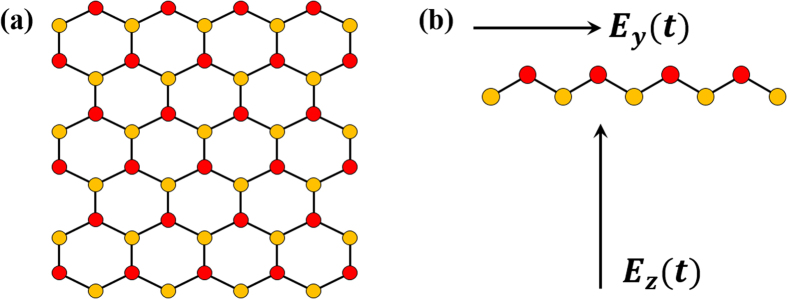
(**a**) The honeycomb lattice and (**b**) buckled structure of silicene. *E*_*y*_(*t*) and *E*_*z*_(*t*) are the time-dependent electric fields along the *y* and *z* directions, respectively.

Silicene consists of a honeycomb lattice of silicon atoms with two sublattices of *A* and *B* sites, as shown in Fig. 1. We consider a silicene sheet in parallel to the *xy* plane. Different from graphene, silicene has a buckled structure, i.e., the two sublattice planes are separated by a small distance *l* ≃ 0.44 Å along the *z* direction^27^. Silicene can be described by the tight-binding model


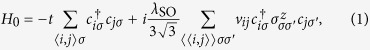


where 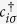 creates an electron with spin polarization *σ* = ↑ or ↓ at site *i*, and 〈*i, j*〉 and 〈〈*i, j*〉〉 run over all the nearest-neighbor and next-nearest-neighbor sites. The first term describes the nearest-neighbor hopping of the electrons with *t* = 1.6 eV. The second term represents the intrinsic spin-orbit coupling with *λ*_SO_ = 3.9 meV, where *v*_*ij*_ = 1 if the next-nearest-neighbor hopping is counterclockwise around a hexagon with respect to the positive *z* axis, and *v*_*ij*_ = −1 if the hopping is clockwise.

For the following calculations, it is sufficient to use the low-energy continuum Hamiltonian, which can be obtained by expanding Hamiltonian (1) around the Dirac points *K* and *K*′ to the linear order in the relative momentum


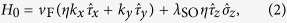


where **k** = (*k*_*x*_, *k*_*y*_) is the relative momentum, *η* = ± correspond to the *K* and *K*′ valleys, and 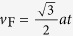 is the Fermi velocity with the lattice constant *a* = 3.86 Å. To drive the quantum pumping, two time-dependent electric fields are applied to the system. One is along the *z* direction of the form 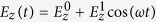 with 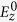 and 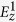 as the amplitudes of the electrostatic component and *ac* component, respectively. The other is an *ac* electric field along the negative *y* direction, *E*_*y*_(*t*) = −*E*_*y*_ cos (*ωt*). By taking the two electric fields into account, the Hamiltonian is rewritten as


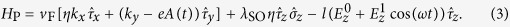


Here −*e* is the electron charge, and *A*(*t*) = *A*_*y*_ sin (*ωt*) is the vector potential of the *ac* electric field along the negative *y* direction with *A*_*y*_ = *E*_*y*_/*ω* and *ω* > 0 being assumed.

### Spin-valley Chern numbers

Within the adiabatic approximation, for a bulk sample, one can obtain for the eigenenergies of Eq. (3) at any given time *t*


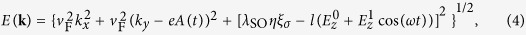


where *ξ*_↑_ = −*ξ*_↓_ = 1. We note that *E*(**k**) depends on valley *η* and spin *σ* only through the product *ηξ*_*σ*_, which has two possible values, *ηξ*_*σ*_ = ±1. It is convenient to consider the whole system as consisting of two subsystems, one with *ηξ*_*σ*_ = 1 and the other with *ηξ*_*σ*_ = −1. For the *ηξ*_*σ*_ = 1 subsystem (i.e., *η* = + and *σ* = ↑, or *η* = − and *σ* = ↓), if 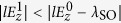, there always exists a finite energy gap between the conduction and valence bands. If 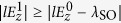, at 
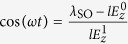
, the conduction and valence bands of the subsystem touch at *k*_*x*_ = 0 and 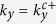 or 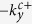. Similarly, for the *ηξ*_*σ*_ = −1 subsystem (*η* = + and *σ* = ↓, or *η* = − and *σ* = ↑), if 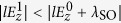, there always exists a finite energy gap between the conduction and valence bands. If 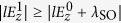, at 
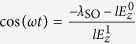
, the conduction and valence bands touch at *k*_*x*_ = 0 and 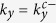 or 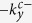. Here,


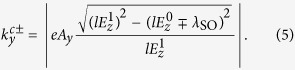


It has been established that the nontrivial topological properties of the system accounting for the spin or valley pumping can be well described by the spin-valley Chern numbers^17^. The topological pumping can be visualized as the quantized spectral flow of the spin-polarized Wannier functions, which originates from the nonzero spin-valley Chern numbers^17^. The spin-valley Chern numbers 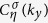 can be defined in the standard way^15^,^16^, on the torus of the two variables *k*_*x*_ ∈ (−∞, ∞) and *t *∈ [0, *T*) with *T* = 2*π*/*ω* as the period. In the present case, because electron spin 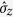 and valley *η* are conserved, the spin-valley Chern numbers are just the first Chern numbers of the occupied electron states of the individual spins and valleys. Specifically, we replace 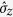 with its eigenvalues *ξ*_↑_ = 1 and *ξ*_↓_  = −1, and rewrite Hamiltonian (3), for given *ξ*_*σ*_ and *η*, into the form 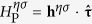, where 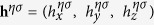 with 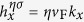, 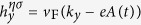, and 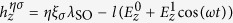. For such a two-band Hamiltonian, the first Chern number of the occupied band is given by^35^ 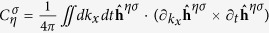, where 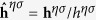 is a unit vector along the direction of **h**^*ησ*^ with 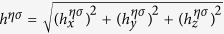. By substituting the expressions for **h**^*ησ*^ into this formula, one can obtain for the spin-valley Chern numbers


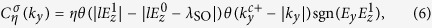


for *ηξ*_*σ*_ = 1, and


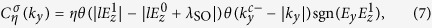


for *ηξ*_*σ*_ = −1, where *θ* (*x*) is the unit step function.

**Figure 2:**
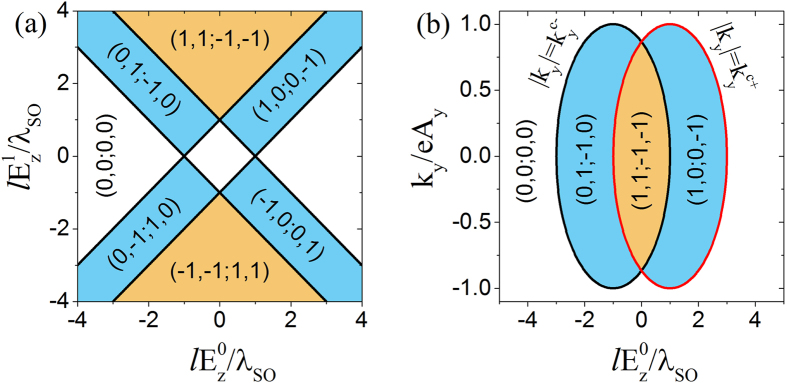
(**a**) The phase diagram of the spin-valley Chern numbers on the normalized 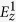 vs normalized 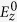 plane for *k*_*y*_ = 0, and (**b**) the phase diagram on the *k*_*y*_ vs 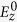 plane for 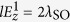. The numbers in the brackets are spin-valley Chern numbers, i.e., 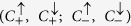. *E*_*y*_ is taken to be positive, and for negative *E*_*y*_, all the spin-valley Chern numbers in the phase diagrams will flip signs.

The phase diagram of the spin-valley Chern numbers for *k*_*y*_ = 0 on the 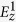 versus 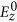 plane is plotted in Fig. 2(a). This phase diagram is mainly determined by the first *θ*-function in Eqs (6 and 7), which sets four straight lines as the phase boundaries, and the second *θ*-function can be considered to be always equal to unity for *k*_*y*_ = 0. The yellow region can be described by the inequations 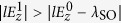 and 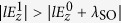. The blue region is given by 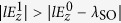 and 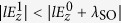, or 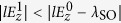 and 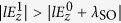. The white region corresponds to 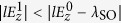 and 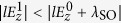. One may notice that on any of the phase boundaries, the band gap always closes at certain time.

A typical phase diagram on the *k*_*y*_ versus 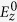 plane for 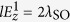 is plotted in Fig. 2(b), where *E*_*y*_ is taken to be positive. The phase diagram can be understood as the superposition of those of the two subsystems of *ηξ*_*σ*_ = 1 and −1, as indicated by Eqs (6 and 7). The phase diagram of the *ηξ*_*σ*_ = 1 subsystem is determined by the boundary 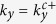, which can be rewritten into the standard form of an ellipse equation 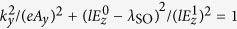, centered at 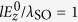 and *k*_*y*_ = 0. The spin-valley Chern numbers of the subsystem take values 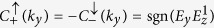 inside the ellipse, and vanish outside the ellipse. Similarly, the phase diagram of the *ηξ*_*σ*_ = −1 subsystem is determined by the boundary 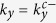, which can be rewritten into the standard form of an ellipse equation 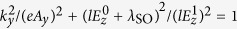, centered at 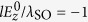 and *k*_*y*_ = 0. The spin-valley Chern numbers of the subsystem take values 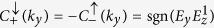 inside the ellipse, and vanish outside the ellipse.

For the convenience to relate the above phase diagram to the spin and valley pumping, we introduce the total valley Chern number 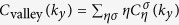 and total spin Chern number 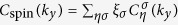. The total charge Chern number 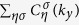 always vanishes and will not be considered. For definiteness, we focus on the case where 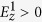, corresponding to the upper half of the phase diagram Fig. 2(a). The opposite case where 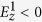, corresponding to the lower half phase diagram, can be understood similarly. When the system is in the yellow region of Fig. 2(a), if 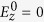, we have 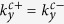, and the spin-valley Chern numbers 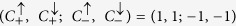 for 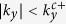 and (0, 0; 0, 0) for 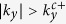, as can be seen from Fig. 2(b). The total valley Chern number *C*_valley_(*k*_*y*_) = 4 for 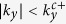, and 0 for 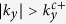. The total spin Chern number *C*_spin_(*k*_*y*_) = 0 for any *k*_*y*_. The system is in the pure valley pumping regime, without pumping spin. If 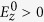, as can be seen from Fig. 2(b), the spin-valley Chern numbers take values (1, 1; −1, −1) for 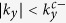, (1, 0; 0, −1) for 
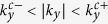
, and (0, 0, 0, 0) for 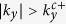. As a result, the electron states with 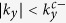 have *C*_valley_(*k*_*y*_) = 4 and *C*_spin_(*k*_*y*_) = 0, and contribute to pure valley pumping, similar to the case for 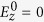. The states with 
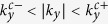
 contribute to both valley and spin pumping, and pump an equal amount of valley and spin quanta per cycle, because *C*_valley_(*k*_*y*_) = *C*_spin_(*k*_*y*_) = 2 in this region. The other states with 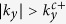 do not contribute to the pumping. Therefore, the system as a whole is in a regime of mixed spin and valley pumping. Each cycle, the system pumps more valley quanta than spin quanta. The case for 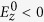 can be analyzed similarly.

When the system is in the blue region of Fig. 2(a), by assuming 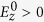 for definiteness, the spin-valley Chern numbers equal to (1, 0; 0, −1) for 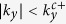, and (0, 0; 0, 0) for 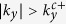. The corresponding total valley Chern number and spin Chern number are *C*_valley_(*k*_*y*_) = *C*_spin_(*k*_*y*_) = 2 for 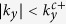, and vanish for 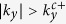. The system is in the spin-valley pumping regime. Different from the spin-valley pumping in the yellow region of Fig. 2(a), each cycle, the system pumps an equal amount of valley and spin quanta. When the system is in the white region of Fig. 2(a), the spin-valley Chern numbers all vanish for any *k*_*y*_, and the system is a trivial insulator.

### Spin Pumping from The Scattering Matrix Formula

The amount of spin and valley quanta pumped per cycle can be conveniently calculated by using the scattering matrix formula^36^,^37^. In the following, we show that the calculated result from the scattering matrix formula is consistent with the above topological description. The spin pumping is more interesting than valley pumping regarding practical applications, and we will focus on the amount of spin pumped per cycle. The valley pumping can be studied similarly by considering an electrode with natural valley degrees of freedom. We consider the pump is attached to a normal electrode, with a potential barrier in between. The total Hamiltonian of the system is taken to be


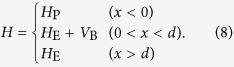


The Hamiltonian *H*_P_ at *x* < 0 for the pump body is given by Eq. (3), and the electrode is taken to be a normal metal with a 2D parabolic Hamiltonian


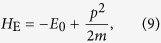


where **p** = (*p*_*x*_, *p*_*y*_) is the 2D momentum, and *E*_0_ and *m* are constant model parameters. In the barrier region, the additional term 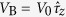 opens an insulating gap of size 2*V*_0_, which accounts for contact deficiencies between the pump and electrode.

The Hamiltonian in the pump is Dirac-like, while in the metal electrode, the Hamiltonian is parabolic. It is well-known that the wavefunction of a parabolic Hamiltonian can not be connected directly to that of a Dirac-like Hamiltonian. To overcome this problem, following Chen *et al*.^17^, we linearize the Hamiltonian Eq. (9) around the Fermi energy before proceeding. When *E*_0_ is sufficiently large, for a given *p*_*y*_, we can linearize the effective 1D Hamiltonian *H*_E_ at the right and left Fermi points 
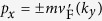
 with 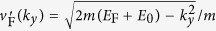. A Pauli matrix 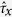 is introduced to describe the right and left-moving branches. To be consistent with the form of the Hamiltonian of the pump and also preserve the time-reversal symmetry, we use *τ*_*x*_ = 1 and −1, respectively, to represent the right-moving and left-moving branches for *η* = 1, and oppositely for *η* = −1. As a result, the Hamiltonian of the electrode becomes


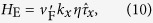


where *k*_*y*_ = *p*_*y*_ and 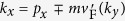 for the right and left-moving branches.

Strictly speaking, the operator 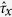 in the electrode has different physical meaning from that in the pump. We notice that in both the pump and electrode, the moving direction (left-moving or right-moving) of a propagating wave is determined by the product 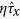. Since when a propagating wave partially transmits across the interface between the pump and electrode, its moving direction does not change, 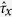 maybe regarded as being the same in the pump and electrode for the transmission process. On the other hand, the difference of the operator 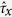 in the pump and electrode alone will cause partial reflection of an incident wave at the interface, even if all the other factors in the pump and electrode match perfectly. Unfortunately, we do not have enough information to accurately parametrize the transmission and reflection amplitudes. To simplify the parametrization, we will omit the difference of the operator 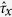 in the pump and electrode, which essentially neglects the reflection effect due to the difference of 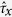. We assume that the reflection effect can be effectively accounted by the potential barrier. This simplification is reasonable, especially in the present system, where the spin pumped per cycle is independent of the material details of the electrode. The pumping effect is usually dominated by small *k*_*y*_, so that we can further approximate 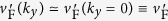, with purpose to minimize the number of adjustable parameters in the model.

Calculation of the number of electrons pumped per cycle amounts to solving the scattering problem for an electron at the Fermi energy incident from the electrode. The Fermi energy will be set to be *E*_F_ = 0, which is in the band gap of the pump. In this case, the incident electron will be fully reflected back into the electrode. In order to obtain the scattering amplitudes, we need to solve the wavefunctions in the three regions. For a spin *σ* electron incident from *η* valley, the wavefunction in the electrode is given by


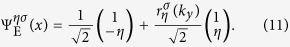


The wavefunctions in the potential barrier and in the pump can be written as


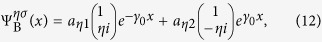



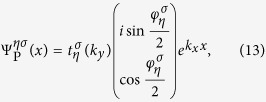


where 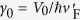, 
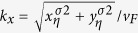
, 
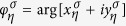
 with 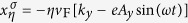 and 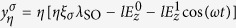. Matching the wavefunctions given in Eqs (11, 12, 13) at *x* = 0 and *x* = *d* by using the boundary conditions 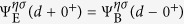 and 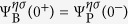, we can obtain for the reflection amplitudes


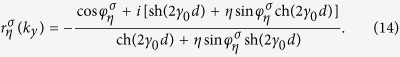


The number of electrons of valley *η* and spin *σ* pumped per cycle at momentum *k*_*y*_ is given by^36^,^37^


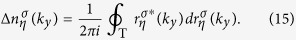


Noting that 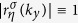 due to full reflection, one can easily identify 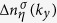 with the winding number of 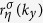 around the origin on the complex plane in a cycle. 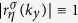 also indicates that with changing the barrier strength *γ*_0_*d*, the trajectory of 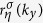 will never sweep through the origin, and so the winding number is invariable. As a result, 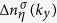 is independent of the barrier strength *γ*_0_*d*. Thus, we can calculate 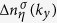 simply by setting *γ*_0_*d* = 0, and the result is valid for any barrier strength. For *γ*_0_*d* = 0, 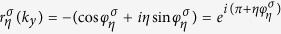, and we can derive Eq. (15) to be 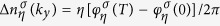.

**Figure 3:**
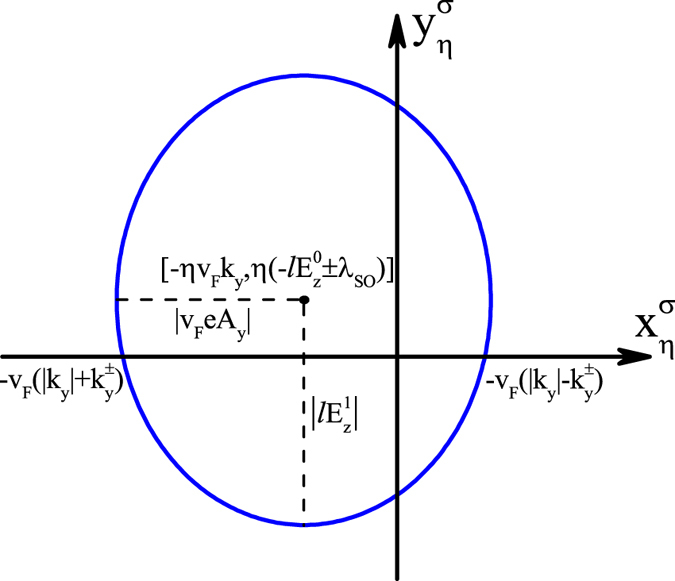
The ellipse trajectory of 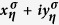 on the complex plane centered at 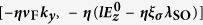, with |*ev_F_A_y_*| and 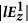 as the semi-major and semi-minor axes oriented along the real and imaginary axes. For the ellipse to surround the origin, two sufficient and necessary conditions must be satisfied. First, the ellipse needs to intersect the real axis. This requires that the semi-minor axis is longer than the distance from the ellipse center to the real axis, namely, 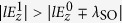 for *ηξ*_*σ*_ = ±1. Second, the two intersecting points between the ellipse and real axis are located at opposite sides of the origin. This results in the condition 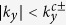 for *ηξ*_*σ*_ = ±1. It is clear that the two conditions are just the two step functions in Eqs (6 and 7).

Because of the periodicity, the increment of 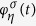 in a period, namely, 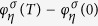, must be integer multiples of 2*π*. From the expression for 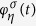 given below Eq. (13), we know that 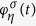 is the argument of 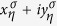. It is clear that the trajectory of 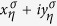 is an ellipse on the complex plane centered at 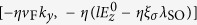, with |*ev*_*F*_*A*_*y*_| and 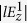 as the semi-major and semi-minor axes oriented along the real and imaginary axes, as shown in Fig. 3. If the ellipse encircles the origin (0, 0), the increment of 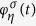 takes value 2*π* or −2*π*, depending on the direction of the trajectory. Otherwise, the increment is 0. The direction of the trajectory is determined by the sign of 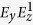. For the ellipse of 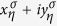 to surround the origin, two sufficient and necessary conditions must be satisfied. First, the ellipse needs to intersect the real axis. This requires that the semi-minor axis is longer than the distance from the ellipse center to the real axis, and so 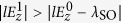 for *ηξ*_*σ*_ = 1, and 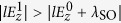 for *ηξ*_*σ*_ = −1. Second, the two intersecting points need to be located at opposite sides of the origin. This results in the condition 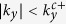 for *ηξ*_*σ*_ = 1, and 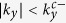 for *ηξ*_*σ*_ = −1. Now it is easy to see that the number of electrons for given valley *η* and spin *σ* pumped per cycle at momentum *k*_*y*_ equals to the spin-valley Chern number 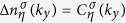.

**Figure 4:**
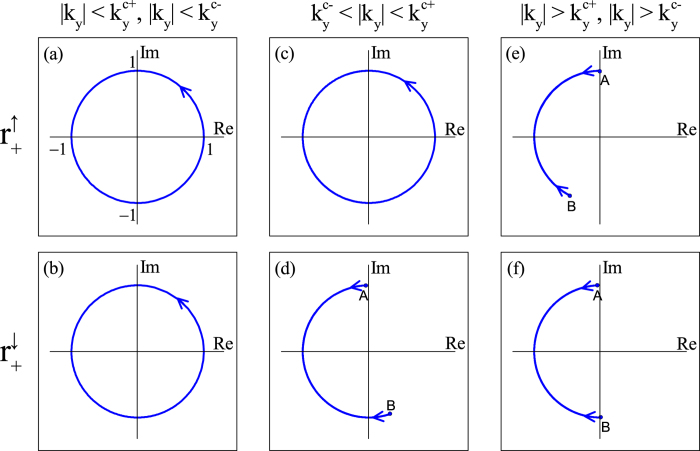
Trajectories of the reflection amplitudes in a cycle on the complex plane, in which only the *η* = + valley is considered. The upper and lower rows correspond to spin *σ* = ↑ and ↓, respectively. In (**a**,**b**), *k*_*y*_ = 0.5* eA*_*y*_, in (**c**,**d**), *k*_*y*_ = 0.8*eA*_*y*_, and in (**e**,**f**), *k*_*y*_ = *eA*_*y*_. The other parameters are set to be *γ*_0_*d* = 1, 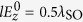 and 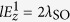, for which the corresponding 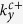 and 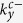 equal to 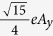 and 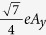, respectively. In (**d**–**f**), the trajectories start from point *A*, travel to *B*, and then return.

To further confirm the above general discussion, in Fig. 4(a–f), we plot the trajectories of 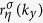 for the *η* = + valley for momentum *k*_*y*_ in different regions. In (a) and (b), the conditions 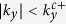 and 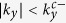 are satisfied, corresponding to the yellow region in Fig. 2(b), and both 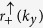 and 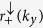 go around the origin counterclockwise once in a cycle. As a result, 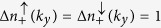, in agreement with the spin-valley Chern numbers 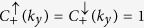. In (c) and (d), we have 
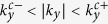
, corresponding to the right blue region in Fig. 2(b), and 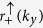 goes around the origin once, but 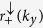 does not. Therefore, 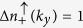 and 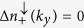, in agreement with 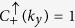 and 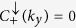. In (e) and (f), we have 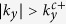 and 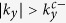, corresponding to the white region in Fig. 2(b), and the winding numbers of 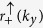 and 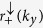 around the origin are zero. Therefore, 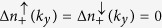, in agreement with 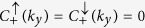. The calculated trajectories of 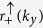 and 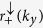 are fully consistent with the spin-valley Chern number description.

Based upon the above discussion, we know that for each *k*_*y*_, the spin pumped per cycle is 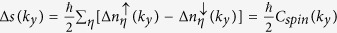. By summing over *k*_*y*_, one can obtain for the total spin pumped per cycle


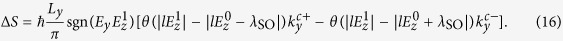


Δ*S* is in scale with the width *L*_*y*_ of the pump, a clear indication that the spin pumping is a bulk effect. If 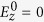, where 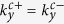, we have Δ*S* = 0. As discussed earlier, the system is in the pure valley pumping regime, without pumping spin. If 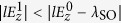 and 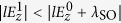, which corresponds to the trivial insulator phase in the white region of Fig. 1(a), we also have Δ*S* = 0. In all other cases, Δ*S* ≠ 0, and the system serves as a topological spin Chern pump.

## Conclusion

We have investigated the topological pumping effect in silicene, modulated by an in-plane and a vertical time-dependent electric field. Using spin-valley Chern numbers to characterize the topological pumping, we find that there exist three quantum pumping regimes in the system, a pure valley pumping regime, a spin-valley pumping regime, and a trivial insulator regime, depending on the strengths of the electrostatic and *ac* components of the perpendicular electric field. The amount of spin pumped per cycle calculated from the scattering matrix formula is fully consistent with the topological description based upon the spin-valley Chern numbers. This work proposed a relatively easy scheme to achieve topological spin or valley Chern pumping. It also demonstrates the fact that bulk topological spin or valley Chern pumping is a characteristic observable effect of various QSH systems, if the material parameters of the QSH systems can be suitably modified with time.

## Additional Information

**How to cite this article**: Luo, W. *et al*. Topological spin and valley pumping in silicene. *Sci. Rep.* **6**, 31325; doi: 10.1038/srep31325 (2016).
